# A bibliometric analysis and visualization of research trends on surgical hip dislocation

**DOI:** 10.1093/jhps/hnac049

**Published:** 2022-12-31

**Authors:** Peng Peng, Tengfei Wei, Weihua Fang, Fangjun Xiao, Xiaoming He, Wei He, Qiushi Wei, Mincong He

**Affiliations:** Guangzhou University of Chinese Medicine, No. 16, Jichang Road, Baiyun District, Guangzhou 510407, P.R. China; Guangzhou University of Chinese Medicine, No. 16, Jichang Road, Baiyun District, Guangzhou 510407, P.R. China; Guangzhou University of Chinese Medicine, No. 16, Jichang Road, Baiyun District, Guangzhou 510407, P.R. China; Guangzhou University of Chinese Medicine, No. 16, Jichang Road, Baiyun District, Guangzhou 510407, P.R. China; Guangdong Research Institute for Orthopedics and Traumatology of Chinese Medicine, No. 261, Longxi Road, Liwan District, Guangzhou 510378, P.R. China; Joint Center, The Third Affiliated Hospital, Guangzhou University of Chinese Medicine, No. 261, Longxi Road, Liwan District, Guangzhou 510378, P.R. China; Guangdong Research Institute for Orthopedics and Traumatology of Chinese Medicine, No. 261, Longxi Road, Liwan District, Guangzhou 510378, P.R. China; Joint Center, The Third Affiliated Hospital, Guangzhou University of Chinese Medicine, No. 261, Longxi Road, Liwan District, Guangzhou 510378, P.R. China; Guangdong Research Institute for Orthopedics and Traumatology of Chinese Medicine, No. 261, Longxi Road, Liwan District, Guangzhou 510378, P.R. China; Joint Center, The Third Affiliated Hospital, Guangzhou University of Chinese Medicine, No. 261, Longxi Road, Liwan District, Guangzhou 510378, P.R. China; Guangdong Research Institute for Orthopedics and Traumatology of Chinese Medicine, No. 261, Longxi Road, Liwan District, Guangzhou 510378, P.R. China; Joint Center, The Third Affiliated Hospital, Guangzhou University of Chinese Medicine, No. 261, Longxi Road, Liwan District, Guangzhou 510378, P.R. China

## Abstract

Surgical hip dislocation (SHD) is a powerful and safe approach used to address pathologic lesions around the hip joint, and therefore, many studies have been conducted in this field. However, no bibliometric studies regarding the global research trend concerning SHD have been studied yet. This study aims to determine the research status in the field of SHD research between 2001 and 2021. The publications related to SHD from 2001 to 2021 were retrieved from the Web of Science Core Collection. Three bibliometric tools were used for this study. The main analyses include publication counts, contributions of countries, institutions, authors, journals and funding agencies, as well as analyses on clustering of references and keywords. In total, 498 articles were identified. The annual publication counts of SHD showed an ascending tendency as a whole. The United States has the most prominent contributions, with the most number of publications and the highest value of H-index. The University of Bern was the organization that produced the most literature. Professors Ganz R, Siebenrock KA, Tannast M, Steppacher SD and Leunig M were the core authors in this field. The most productive journal was *Clinical Orthopaedics and Related Research.* Burst keyword detection suggested that the following research directions, including ‘surgical hip dislocation’, ‘outcome’, ‘fixation’ and ‘pain’, are considered the research hotspots and deserve more attention. In conclusion, this is the first bibliometric analysis that provides a comprehensive overview of SHD research, which may assist investigators in exploring new directions for this technique.

## INTRODUCTION

Surgical hip dislocation (SHD) is a powerful and safe approach used to address all pathologic lesions around the hip joint. The technique was developed and popularized by Ganz and colleagues, which allowed for safe dislocation of the hip without compromising blood supply to the femoral head [1]. With this approach, direct visualization of the hip is obtained while minimizing trauma to the abductor musculature [1, 2]. The most common drawbacks of this technique include the likelihood of trochanteric nonunion and low-grade heterotopic ossification [3, 4]. However, it has been widely used in hip diseases including femoroacetabular impingement (FAI), periarticular trauma, Legg–Calvé–Perthes disease (LCPD), slipped capital femoral epiphysis (SCFE), benign lesions of the hip joint and osteochondral lesions [5–12]. In the past two decades, the number of studies on SHD has been growing, and indications are evolving as an understanding of hip pathology improves. Thus, it is necessary to analyze the development trends and research status of SHD, which may provide help for the expansion of indications and the improvement of this technique.

Bibliometric analysis is a useful method that combines statistical methods with data visualization technology for qualitative and quantitative analyses of academic publications within a certain field [13]. Over the years, it has been widely used in orthopedic fields to estimate the research trends of orthopedic disorders and surgical approaches, such as hip fracture [14], knee osteoarthritis [15], developmental dysplasia of the hip [16] and keen and hip arthroplasty [17].

Therefore, this study aimed to use the bibliometric method to identify the development trends and research status of SHD. Our study may provide helpful information to understand and promote this technique.

## MATERIALS AND METHODS

### Data acquisition and retrieval strategies

**Fig. 1.**
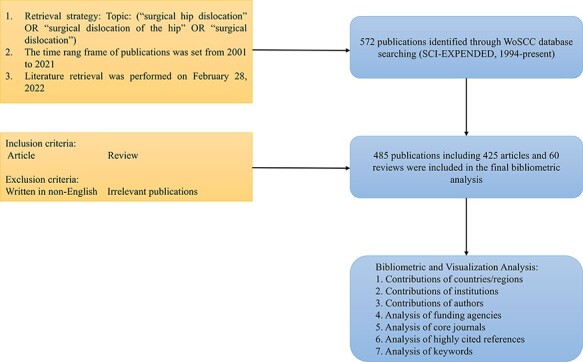
Flowchart for the selection of literature included in this study.

We obtained literature from the Web of Science Core Collection (WoSCC). Literature retrieval was performed within 1 day (17 February 2022). The detailed retrieval strategy was as follows: TS = (‘surgical hip dislocation’ OR ‘surgical dislocation of the hip’ OR ‘surgical dislocation’). We included publications from 2001 to 2021 (31 December 2021), and the language was restricted to English. The document types were limited to original articles and reviews. Figure 1 presents the literature search and selection process.

### Data extraction and collection

We manually excluded the documents of irrelevant publications. All retrieved literature was downloaded and exported in text format. We collected information from the selected articles including titles, authors, affiliations, countries, keywords, journal, publication year, funding agencies, average citation per item (ACI) and H-index. The H-index was a broad measure of the impact of a researcher’s scientific contributions [18]. Microsoft Office Excel 2019 (Microsoft Corporation, Redmond, WA, USA) was used to perform data entry, cleaning and descriptive statistical analysis.

### Bibliometric and visualized analysis

In this study, VOSviewer (version 1.6.16) [19], CiteSpace V (version 5.7. R5) [20] and an online analytical platform (https://bibliometric.com/) were used to perform this bibliometric analysis. In this study, we used the default parameters in CiteSpace and VOSviewer. VOSviewer was used to visualize the co-citation of journals, coauthorship of countries and keyword co-occurrence. The size of the nodes reflected the number of publications, citations or occurrences. The links between nodes represented the associations, including coauthorship or co-citation. The weighted total link strength (TLS) was used to measure the strength of the links of the selected nodes.

CiteSpace was utilized to conduct cooperation and co-citation analyses of institutions or authors, the dual-map overlay of scientific journals and burst keywords. Betweenness centrality (BC) is a crucial parameter that could measure the scientific importance of the nodes in the network, and nodes with high BC (BC ≥ 0.1) are usually indicated by purple rings and also connect more links. In terms of the clusters view map, cited authors with similar categories were gathered in a cluster. The bursts of keywords are often used to detect new research trends in the field. Through detailed analysis using CiteSpace, we have selected the top 10 keywords with the strongest citation bursts.

## RESULTS

### Global publication and citation trend

**Fig. 2.**
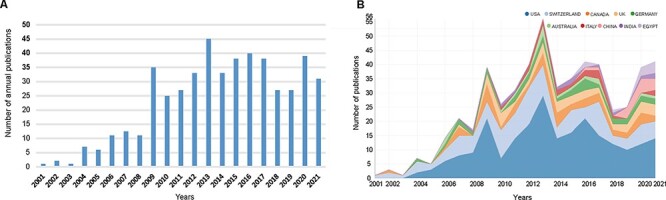
(A) The number of annual publications and citations on SHD research from 2001 to 2021. (B) The annual number of publications in the top 10 most productive countries from 2001 to 2021.

In total, 485 publications (425 articles and 60 reviews) were included in this study (Fig. 1). Trends in the number of annual publications are presented in Fig. 2A. Despite the appearance of the volatility to decrease at some time points, the annual number of publications related to SHD showed an ascending tendency as a whole and reached its peak in 2013 with a total of 45 documents. The number of publications increased by 3100% from 2001 to 2021, and almost 33.8% of them (164 papers) were published over the last 5 years.

### Contributions of countries/regions

**Fig. 3.**
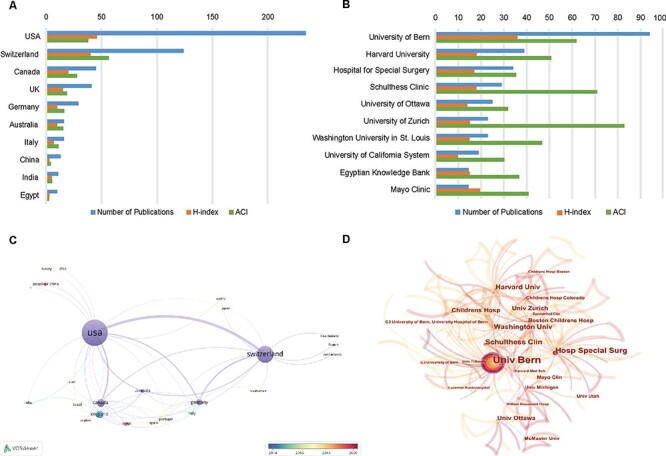
(A) The top 10 countries with the most publications related to SHD research. (B) The top 10 institutions with the most publications related to SHD research. (C) The coauthorship map of countries/regions involved in SHD research (generated by VOSviewer). (D) The cooperation network map of institutions involved in SHD research (generated by CiteSpace).

The variation trend in the annual publication numbers from the top 10 productive countries from 2001 to 2021 was illustrated in Fig. 2B. A total of 39 countries/regions contributed to the field of SHD research and the top 10 most productive countries are shown in Fig. 3A. The United States had the largest number of publications, with 224 articles published (46.2%), followed by Switzerland (121, 24.9%), Canada (45, 9.3%), the United Kingdom (41, 8.5%) and Germany (29, 6.0%). The United States also had the highest H-index (H-index = 46), followed by Switzerland (H-index = 40) and Canada (H-index = 20). Switzerland had the highest average number of citations (56.63), followed by the United States (38.03), Canada (27.8) and the United Kingdom (18.71). The visualization map of research collaboration among countries/regions is presented in Fig. 3C. In this network, the United States collaborated most closely with Switzerland and Canada, and the top three with the largest TLS were listed as follows: the United States, Switzerland and Canada.

### Contributions of institutions

In terms of research institutions, the top 10 are specifically listed in Fig. 3B. Of these, five were US institutions, and three were Switzerland institutions. Among them, the University of Bern holds the largest number of publications, followed by Harvard University and Hospital for Special Surgery. The H-index of 36 in the University of Bern exceeded other institutions, ranking first. In terms of ACI, the University of Zurich had the most average number of citations (82.83). The institution network map of the SHD research was generated by CiteSpace and is presented in Fig. 3D. The University of Bern, Hospital for Special Surgery and Harvard University were the top three institutions in terms of centrality.

### Contributions of authors

**Fig. 4.**
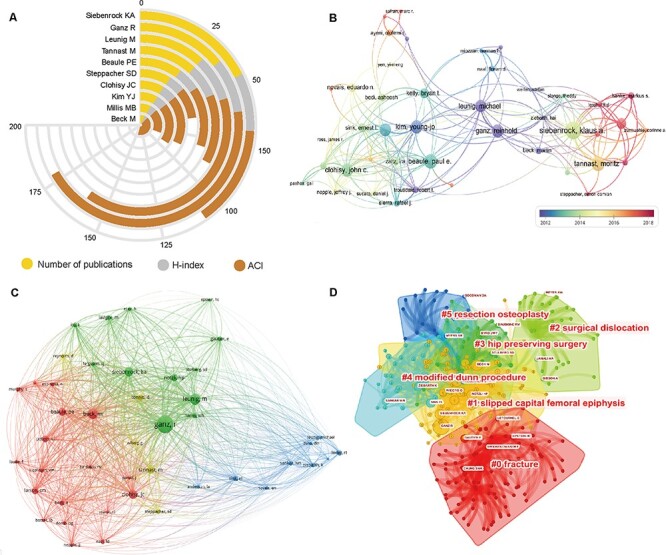
(A) The top 10 most productive authors in SHD research. (B) Author coauthorship overlay visualization map generated by VOSviewer. (C) Network visualization map of author co-citation analysis generated by the VOSviewer. (D) In the cluster map, cited authors with similar categories were gathered in a cluster.

The top 10 authors who contributed most are presented in Fig. 4A. Siebenrock KA was the author with the most publications of 47, followed by Ganz R, Leunig M, Tannast M, Beaule PE and Steppacher SD. Figure 4B is an overlay visualization map for author coauthorship analysis with minimum publications of five. In the network map, Siebenrock KA, Tannast M and Steppacher SD were located at the central position of the cooperating clusters with the largest TLS. Based on the co-citation analysis performed with VOSviewer (Fig. 4C), we defined ‘core author’ as one who had acquired at least 50 citations. The top three authors with the largest TLS were Ganz R, Beck, M and Leunig M. Meanwhile, the co-citation relationships between authors were analyzed by CiteSpace via creating network visualization maps. As for the cluster view of the co-citation map (Fig. 4D), the silhouette value of clusters #0 to #5 was from 0.875 to 0.958, suggesting good homogeneity. Research categories of authors were divided into six clusters, including ‘fracture’ (#0), ‘slipped capital femoral epiphysis’ (#1), ‘surgical dislocation’ (#2), ‘hip preserving surgery’ (#3), ‘modified Dunn procedure’ (#4) and ‘resection osteoplasty’ (#5).

### Analysis of funding agencies

**Fig. 5.**
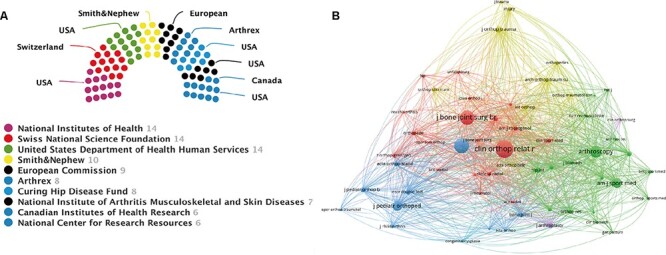
(A) Top 10 related funding agencies for the support of SHD research. (B) Journal co-citation analysis by using VOSviewer.

The National Institutes of Health, Swiss National Science Foundation and United States Department of Health Human Services all funded the most publications (14; 2.8%), followed by the Smith & Nephew (10; 2.0%) and the European Commission (9; 1.8%) (Fig. 5A). Of these funding agencies, five funding institutions in the United States provided funding for the SHD research. The remaining five funding institutions were located in Switzerland, Smith & Nephew, Arthrex, Canada and European Union, respectively.

### Analysis of core journals

The top 10 active journals published 261 articles on SHD, accounting for 53.8% of all 498 publications. Table I shows the information on the top 10 journals. Of these, *Clinical Orthopaedics and Related Research* (65, 13.4%) had the highest number of outputs, followed by *Journal of Pediatric Orthopaedics* (38, 7.8%) and *Hip International* (31, 6.1%). *American Journal of Sports Medicine* has the largest impact factor of 6.202. According to the Journal Citation Reports (JCR) 2020 standards, the top 10 most prolific journals were classified as Q1 in 5, Q2 in 2 and Q4 in 3. VOSviewer software was used to analyze the co-citation of journals. As shown in Fig. 5B, 59 journals with a minimum of 30 citations were included. The top three journals with the largest TLS were listed as follows: *Clinical Orthopaedics and Related Research, Journal of Bone and Joint Surgery—American Volume* and *Journal of Bone and Joint Surgery—British Volume*.

**Table I.** The top 10 journals with the most publications in SHD research

*Rank*	*Journal title*	*Counts*	*Percentage (N/485)*	*IF (2020)*	*JCR (2020)*	*H-index*	*ACI*
1	*Clinical Orthopaedics and Related Research*	65	13.4	4.176	Q1	33	75.57
2	*Journal of Pediatric Orthopaedics*	38	7.8	2.324	Q2	13	20.34
3	*Hip International*	31	6.1	2.135	Q4	12	13.68
4	*Journal of Bone and Joint Surgery—American Volume*	28	5.8	5.284	Q1	17	62.07
5	*Bone & Joint Journal*	22	4.5	5.082	Q1	12	19.73
6	*American Journal of Sports Medicine*	20	4.1	6.202	Q1	15	60.65
7	*Arthroscopy*: *The Journal of Arthroscopic and Related Surgery*	18	3.7	4.772	Q1	17	56.68
8	*Journal of Orthopaedic Trauma*	14	2.9	2.512	Q4	7	11.93
9	*Journal of Hip Preservation Surgery*	13	2.7	1.872	Q4	4	3.38
10	*International Orthopaedics*	12	2.5	3.075	Q2	6	15

.

### Analysis of highly cited references

Table II lists the top 10 most cited articles. The highest and lowest numbers of citations of an article were 915 and 188, respectively. Articles from Switzerland accounted for five, and articles from the United States accounted for four. All these studies were published between 2001 and 2009, and all of them acquired more than 180 citation times. Supplementary Fig. S1 illustrated the top 20 references with the strongest citation bursts. In this map, the blue lines indicated the time interval, and the red part represented the time period when the reference burst occurred. Among them, the reference with the strongest burst value was published by Beck *et al.* [21], followed by Peters *et al.* [22] and Espinosa *et al.* [23]. Notably, all three references investigated the application of SHD for the treatment of FAI.

**Table II.** The top 10 most cited works of literature in SHD research

*Rank*	*Article title*	*Citations*	*Author*	*Journal title*	*Year*
1	Surgical dislocation of the adult hip—a technique with full access to the femoral head and acetabulum without the risk of avascular necrosis	915	Ganz, R	*Journal of Bone and Joint Surgery—British Volume*	2001
2	The etiology of osteoarthritis of the hip—an integrated mechanical concept	723	Ganz, R	*Clinical Orthopaedics and Related Research*	2008
3	Anterior femoroacetabular impingement: part II. Midterm results of surgical treatment	525	Beck, M	*Clinical Orthopaedics and Related Research*	2004
4	Anterior femoroacetabular impingement: part I. Techniques of joint preserving surgery	337	Lavigne, M	*Clinical Orthopaedics and Related Research*	2004
5	Treatment of femoro-acetabular impingement: Preliminary results of labral refixation	336	Espinosa, N	*Journal of Bone and Joint Surgery—American Volume*	2006
6	Clinical presentation of patients with symptomatic anterior hip impingement	292	Clohisy, JC	*Clinical Orthopaedics and Related Research*	2009
7	Debridement of the adult hip for femoroacetabular impingement	256	Murphy, S	*Clinical Orthopaedics and Related Research*	2004
8	Quality of life following femoral head-neck osteochondroplasty for femoroacetabular impingement	204	Beaule, PE	*Journal of Bone and Joint Surgery—American Volume*	2007
9	Open surgical dislocation versus arthroscopy for femoroacetabular impingement: a comparison of clinical outcomes	198	Botser, IB	*Arthroscopy*	2011
10	Hip damage occurs at the zone of femoroacetabular impingement	188	Tannast, M	*Clinical Orthopaedics and Related Research*	2008

### Keyword analysis of research hotspots

**Fig. 6.**
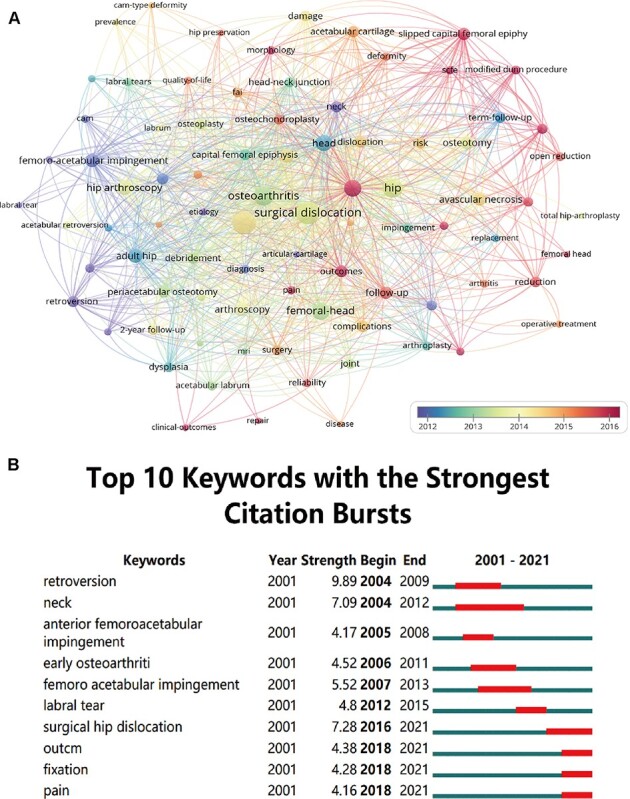
(A) The overlay visualization map of the keyword co-occurrence analysis using VOSviewer. (B) The top 10 keywords with the strongest citation bursts from 2001 to 2021 (generated by CiteSpace).

A total of 1197 keywords were extracted from 498 publications. In addition, we provided an overlay visualization map of co-occurrence keywords (Fig. 6A). Different colors were applied for each keyword according to their average appearing year in articles. Keywords such as outcomes, modified Dunn procedure, slipped capital femoral epiphysis and pain have emerged recently, which indicated that this topic may continue to be hotspots of the SHD research field.

Moreover, burst keywords were regarded as another important indicator of research hotspots, predicting the emerging tendencies to a certain extent. In this study, we applied the burst detection algorithm to extract keywords for SHD research. Figure 6B illustrates the top 10 keywords with the strongest citation bursts from 2001 to 2021. The main research hotspots are FAI, SHD, fixation and pain. Among the whole list with the strongest citation bursts, ‘retroversion’, ‘surgical hip dislocation’, ‘neck’ and ‘femoroacetabular impingement’ were the top four keywords with the strongest burst strength (9.89, 7.28, 7.09 and 5.52, respectively). Notably, we also found that the citation burst time of keywords including ‘surgical hip dislocation’, ‘outcome’, ‘fixation’ and ‘pain’ has continued to 2021, and the bursts are still ongoing.

## DISCUSSION

In this study, we presented a comprehensive overview of the current status, research hotspots and theme trends in SHD research. Over the last two decades, the number of publications in SHD showed an ascending tendency as a whole. The United States has the largest number of publications and the highest value of H-index, indicating that this country contributes most to this field. Switzerland has also made a significant contribution in this field, reflecting the highest value of the average number of citations. Some countries such as Canada, the United Kingdom and Germany have also played an important role based on their publication outputs. Nevertheless, most countries in Africa and Southeast Asia do not contribute to this field, and their lower economic level may help explain this phenomenon.

Among the top 10 institutions, 5 are from the United States, 3 are from Switzerland, 1 is from Canada and 1 is from Egypt. These results implied that first-class research institutions are critical for improving a country’s academic standards. The collaboration visualization map indicated that the interinstitutional collaboration was relatively low and mainly scattered in US and Switzerland institutions. Although some Asian countries have contributed in the number of publications, there was no cooperation network among institutions in these regions. Therefore, there is an urgent need to improve academic collaborations in different institutions.

Cooperation and co-citation analysis could provide information on the existing partnerships of the core authors in the field of SHD research. Our results showed that Ganz R, Siebenrock KA, Tannast M, Steppacher SD and Leunig M were the core authors in this field. For example, Ganz R from the University of Bern published a large number of papers with the highest value of H-index and ACI in this field. The chief contribution of Professor Ganz R was proposing and popularizing the technique of SHD [1]. Professor Siebenrock KA has conducted many of clinical research regarding the treatment of various hip diseases including FAI, acetabular fractures, SCFE and avascular necrosis (AVN) of the femoral head [24–26]. Professor Leunig M is one of the top experts in the field of SHD from the Schulthess Clinic Zurich. The main contribution of him was providing a great deal of clinical research data on FAI [27, 28]. Furthermore, in the clustering analysis, ‘fracture’, ‘slipped capital femoral epiphysis’, ‘surgical dislocation’, ‘hip preserving surgery’, ‘modified Dunn procedure’ and ‘resection osteoplasty’ contained the largest authors group, which indicated that these research topics obtained the most attention.

Keywords can intuitively and accurately reflect the research topic and core content of an article. The research directions and hotspots in the field of SHD were identified using the co-occurrence analysis. From the results of keyword co-occurrence analysis, the focus of research on SHD includes the application of SHD in the treatment of various hip diseases and follow-up studies on clinical outcomes and complications.

The common indications for SHD are FAI, LCPD, SCFE, periarticular trauma, osteochondral lesions and benign lesions of the hip joint. Taking FAI as an example. The indications for the treatment of FAI with an SHD approach include suspected extraarticular impingement and lesions that are difficult to address arthroscopically [29]. Advantages of an SHD approach to FAI include the ability to visualize the intra-articular pathologic alterations, allowing an effective elimination of the bony and soft tissue distortions, causing clinical symptoms [30]. Overall, SHD remains a favorable surgical approach to address patients with FAI [31, 32]. For another example, the modified Dunn osteotomy using SHD is a good choice for patients with moderate and severe SCFE, allowing anatomical restoration of proximal femur, direct inspection, preservation of physical blood supply and inspection of intra-articular pathology [33, 34].

Overall, clinical outcomes demonstrate that SHD is a successful and safe procedure that improved pain, range of motion and clinical outcomes in FAI, LCPD, SCFE, periarticular trauma and osteochondral lesions [5, 21, 34, 35–37]. A multicenter study analyzed the incidence of complications associated with SHD and reported that many of the complications were clinically unimportant heterotopic ossification [3]. There were no cases of femoral head osteonecrosis or femoral neck fracture in this large cohort [3]. Freccero *et al.* provided clear radiographic evidence that SHD may be performed without causing AVN [38]. A retrospective study reviewed 29 patients with symptomatic LCPD who underwent the SHD approach and reported that no patients had osteonecrosis, implant failure, deep infection or nonunion [39]. In the treatment of SCFE using a modified Dunn, the reported rates of AVN vary depending on the series from 0% to 26% [9, 40].

### Limitations

This research still has some limitations. First, an unavoidable limitation of the bibliometric analysis was the potential for incomplete searches of studies due to the restriction of the search terms. This may partially affect the precision of the results but is unlikely to change the final conclusions. Second, bibliometric analyses included only the publications from the WoSCC database and neglected other large medical databases, which could miss a few relevant articles. Third, only English-based publications were included in the final analysis, which may cause language bias. Additionally, most of the results of this study are based on machine algorithms, which are slightly insufficient in artificial induction.

## CONCLUSION

This study presented a comprehensive overview of the current status and research hotspots in SHD research between 2001 and 2021 and predicted future theme trends in this field. Over the last two decades, the number of publications in SHD showed an ascending tendency as a whole. Clinical outcome, complications and their applications in various hip diseases have been research hotspots in the field of SHD in recent years. This study may assist investigators in exploring new directions for this technique.

## Supplementary Material

hnac049_Supp

## Data Availability

The data underlying this article will be shared on reasonable request to the corresponding author.
