# Supplementary material for: A bibliometric analysis and visualization of research trends on surgical hip dislocation
Source: J Hip Preserv Surg. 2022 Dec 31;10(1):8–16. doi: 10.1093/jhps/hnac049 (PMC10234387; doi:10.1093/jhps/hnac049)
Supplement: hnac049_Supp [file hnac049_supp.zip › suppl_data/Supplementary Fig.docx]

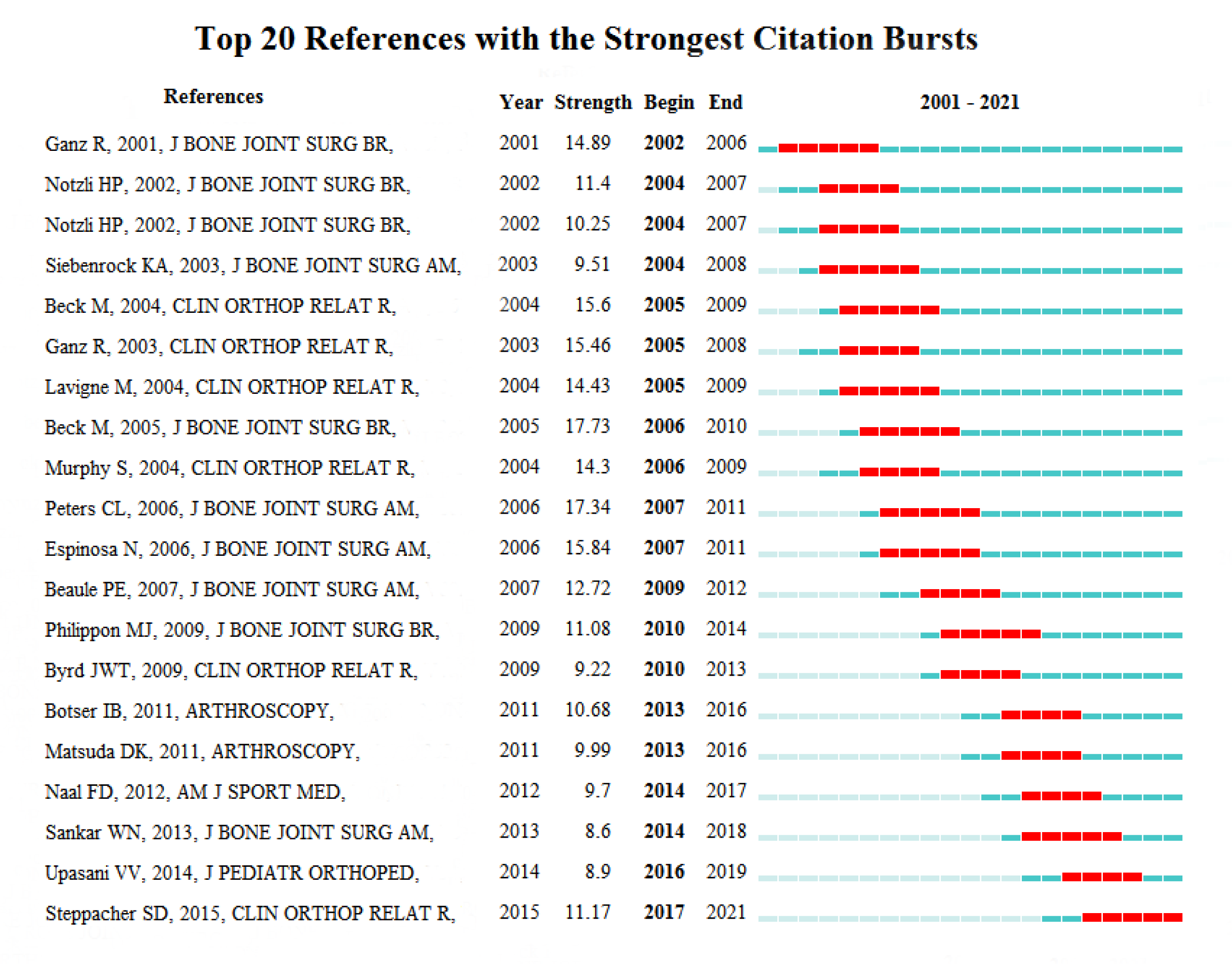


**S[upplementary Fig. S1](https://www.ncbi.nlm.nih.gov/pmc/articles/PMC9174950/#s11)** The top 20 references with the strongest citation bursts from 2001 to 2021 (generated by CiteSpace).
